# The use of soluble fibre for the management of chronic idiopathic large-bowel diarrhoea in police working dogs

**DOI:** 10.1186/s12917-021-02809-w

**Published:** 2021-03-02

**Authors:** J. C. Alves, A. Santos, P. Jorge, A. Pitães

**Affiliations:** 1 Guarda Nacional Republicana (Portuguese Gendarmerie), Lisbon, Portugal; 2 MED – Mediterranean Institute for Agriculture, Environment and Development, Instituto de Investigação e Formação Avançada, Universidade de Évora, Pólo da Mitra, Ap. 94, 7006-554 Évora, Portugal; 3 Vale Referrals, the Animal Hospital, Stinchcombe, Dursley, Gloucestershire GL116AJ UK

**Keywords:** Dog, Diarrhoea, Psyllium

## Abstract

**Background:**

Chronic intermittent or persistent diarrhoea is a common condition in dogs and may be a reflex of gastrointestinal or non-gastrointestinal disorders. Besides diarrhoea, many athletes experience other gastrointestinal symptoms. Dietary fiber can help normalize colonic motility and transit time, support normal gastrointestinal microflora growth and provide fuel for colonocytes. This study aimed to evaluate dietary supplementation effectiveness with psyllium husk in police working dogs with chronic large-bowel diarrhoea. Twenty-two animals were selected. Concurrent conditions were ruled out through complete blood count and serum biochemistry. Fecal *Clostridium* and *Salmonella* were also screened. A soluble fiber, psyllium husk, was added to the diet at the dose of 4 tablespoons/day for 1 month. A daily log of fecal characteristics (type, frequency, and color) was maintained during the supplementation month and for an additional month, without supplementation.

**Results:**

Response to treatment was classified as “very good” in 50% of animals, “good” in 40% of animals, and “poor” in 10% of cases. During the month of psyllium husk supplementation, defecation frequency decreased from 3.5 to 2.9 times a day, with 90% of animals showing consistent stools regularly and registering a mean increase of 2 kg in body weight. Beneficial effects were still observed during the second month, without psyllium husk supplementation.

**Conclusion:**

Psyllium husk can be useful in the management of chronic large-bowel diarrhoea in working dogs, which exhibited lower defecation frequency, improved stool consistency, and gained weight. Effects were felt beyond the supplementation period. Alternative approaches for non-responsive cases need to be evaluated.

## Background

Chronic intermittent or persistent diarrhea is a common condition in dogs and may reflect gastrointestinal or non-gastrointestinal disorders [1]. Diarrhea is considered chronic if present for at least 2 days/week over at least 4 weeks [2, 3]. Chronic idiopathic diarrhoea is usually characterized by an increased frequency of defecation, usually by excess faecal mucus, and, to be classified as such, other conditions must be ruled out. Chronic large-bowel diarrhoea is commonly caused by various factors, ranging from whipworms, dietary indiscretion, *Clostridium perfringens* enterotoxicosis, neoplasia, and irritable bowel disease [4–6].

While laboratory findings usually show normal complete blood counts and serum biochemistry, systemic diseases must be ruled out, and faecal examination should be conducted to dismiss whipworms, *Salmonella* spp. and *Campylobacter* spp. [2, 3]. Many patients show no physical abnormalities during the examination, but some may present with weight loss and poor body condition score [5].

In humans, runner’s diarrhoea has also been described, which affects from 30 to 90% of athletes, with a toll on performance, mainly due to mechanical, ischemic, and nutritional factors [7]. Besides diarrhoea, many athletes experience gastrointestinal symptoms, from nausea, cramping, and bloating [8]. Interestingly, this phenomenon, which affects athletes other than runners, seems to occur more at rest (after exercise) than during exercise itself [9]. A similar phenomenon may be present in working dogs.

Dietary fiber can help normalize colonic motility and transit time, support normal gastrointestinal microflora growth and provide fuel for colonocytes. Soluble fibers can be added to a normal diet to improve the faeces’ consistency, as they have great water-holding capacities, forming gels in water. They can also influence the large intestine microflora constitution and increase colonic weight and surface area, increasing absorptive capacity [5, 10, 11]. This microflora also ferments soluble fiber and produces volatile fatty acids that promote colonocyte regeneration and may improve recovery from diarrhoea [12–14]. Psyllium husk is a mixed fiber source, commonly used in commercial pet foods and supplementation outside diet, and is particularly useful in working dogs with stress/working diarrhoea [2, 11, 15]. Weekly recording of body weight, body condition score (BCS), and stool evaluations are useful for assessing patients with chronic colitis [5].

This study aimed to evaluate if the supplementation with psyllium husk would help manage chronic idiopathic large-bowl diarrhoea in police working dogs.

## Results

The sample included 22 animals, of both sexes (all intact, 17 males and 5 females), with a mean age of 4 ± 1.4 years, bodyweight of 27.6 ± 5.2 kg, and a body condition score of 4/9. Four breeds were represented: Belgian Malinois Shepherd Dogs (BM, *n* = 12), German Shepherd Dogs (GSD, *n* = 9), and Dutch Shepherd Dog (DSD, *n* = 1). Of the animals enrolled in the study, no abnormalities were observed during the physical examination, and CBC results and serum biochemistry were also normal in all animals. Overall CBC results and serum biochemistry registered can be observed in Table 1.

**Table 1:** Results of complete blood count results and serum biochemistry registered

Parameter	Mean Value	SD	Reference Value
RBC (× 10^12^/L)	6.84	0.92	5.5–8.5
Hemoglobin (g/L)	153.9	0.00	120–180
Hematocrit (Proportion of 1.0)	0.46	0.06	0.37–0.55
Total white blood cells (×10^9^/L)	9.77	3.93	6.0–17.0
Lymphocytes (×10^9^/L)	2.06	1.02	1.0–4.8
Monocytes (×10^9^/L)	0.69	0.39	0.2–2
Neutrophils (×10^9^/L)	6.68	0.36	3.0–11.8
Eosinophils (×10^9^/L)	0.31	0.36	0.1–1.3
Basophils (×10^9^/L)	0.03	0.01	0.0–0.5
Platelets (×10^9^/L)	295.94	59.84	200–500
Urea (mmol/L)	12.89	5.03	5.34–14.28
Creatinine (μmol/L)	73.37	30.06	35.36–123.76
AST (μkat/L)	0.71	0.26	0.17–0.67
ALT (μkat/L)	5497.80	2684.95	1122.0–7854.0
Bilirrubine (μmol/L)	1.03	0.68	1.71–5.13
Amylase (μkat/L)	9.89	4.99	3.34–21.54
Lipase (μkat/L)	0.56	0.19	< 8.35
PT (g/L)	588.68	3,80	55–75
ALB (g/L)	30.06	2.88	23–31
Colesterol (mmol/L)	4.79	0.98	2.85–8.13
Ca^2+^ (mmol/L)	2.28	0.27	2.23–0.28
Na^+^ (mmol/L)	145.41	1.73	135–146
K^+^ (mmol/L)	5,02	0.4	3.3–5
Cl^−^ (mmol/L)	111.91	2.66	94–113
P (mmol/L)	4,92	2.79	2.5–6

Faecal flotation and screenings were also negative. All animals showed continuous diarrhoea and increased defecation frequency, with a mean value of 3.5 times a day (range 3–6 times a day). Also, 37.5% of cases presented permanent decreased faecal consistency. For the remaining animals, faecal consistency showed a decreasing tendency with subsequent defecations. All animals presented faecal mucus. As a rule, animals included in this study can be described as extremely active, both during work and when kennelled, with very high drive. As they are housed in a facility with many dogs, environmental stress factors are also present.

Response to treatment was classified as “very good” in 50% of animals, “good” in 40% of animals, and “poor” in 10% of cases. During the month of psyllium supplementation, defecation frequency decreased from 3.5 to 2.9 times a day (*p* < 0.01), with 90% of animals showing consistent stool regularly (scores 2–5 in the Bristol stool chart). An increase in faecal bulk was observed. A mean increase of 2 kg in body weight was observed (range 0.4–4.8 kg, *p* = 0.01). During the second month of monitoring, without psyllium supplementation, defecation frequency showed a very mild increase to a mean value of 3.0 times a day, with 90% of animals still exhibiting consistent stools, with only sporadic episodes of diarrhoea. Defecation frequency returned to values near initial evaluation only in week 3 without psyllium supplementation (*p* = 0.06). The overall distribution of the relative frequency of faecal scores can be observed in Table 2. During this period, response to treatment was still classified as very good in 50% of animals, good in 40% of animals, and poor in 10% of cases. A mean increase of 0.6 kg in body weight was still observed (range − 0.7-2.9 kg, *p* = 0.06). No additional medication was administered to any of the dogs enrolled.

**Table 2:** Distribution of relative frequency of faecal scores according to the Bristol stool chart with and after supplementation with soluble fiber

Score	T0	1st month					2nd month				
		week 1	week 2	week 3	week 4	week 5	week 1	week 2	week 3	week 4	week 5
**1**	0,0	0,0	0,0	0,0	0,0	0,0	0,0	0,0	0,0	0,0	0,0
**2**	0,0	0,0	28,6	28,6	40,0	28,6	25,7	25,7	31,4	25,7	25,7
**3**	14,3	37,1	31,4	28,6	20,0	25,7	28,6	42,9	31,4	34,3	37,1
**4**	28,6	28,6	5,7	22,9	14,3	31,4	22,9	20,0	8,6	11,4	11,4
**5**	14,3	5,7	31,4	14,3	20,0	14,3	20,0	11,4	25,7	20,0	17,1
**6**	42,9	28,6	2,9	5,7	5,7	0,0	2,9	0,0	2,9	8,6	8,6
**7**	0,0	0,0	0,0	0,0	0,0	0,0	0,0	0,0	0,0	0,0	0,0

## Discussion

In this study, daily supplementation with psyllium husk improved clinical signs in police working dogs with chronic diarrhoea, decreasing defecation frequency and improving stool consistency. Treated animals also exhibited a weight gain. These beneficial effects were still felt in the second month of monitoring without psyllium supplementation for most animals.

In a previous study regarding the management of chronic large-bowel diarrhoea laboratory results are usually normal, with only mild abnormalities identified in 37.8% of cases: high ALP, a slight increase in ALT, slight hypoalbuminemia, lymphocytosis, eosinophilia, lymphopenia, and slight neutropenia [2]. Other reports point out that working dogs, in particular, typically have higher values of ALP, which, together with lipase, can help determine the presence of pancreatitis in dogs [16–19]. A lack of recovery and the chronicity of the condition is frequently associated with anaemia and severe hypoalbuminemia [1]. Our results are in line with these reports, as no abnormalities were observed during the physical examination, and CBC scores and serum biochemistry were also normal.

Various personality traits and stressful factors have been described and associated with chronic large-bowel diarrhoea in dogs. Dogs with chronic large-bowel diarrhoea have been described as nervous and high-strung, accounting for 37.8% of cases in a review study [2]. This high-strung characteristic can describe all of the dogs included in this study. In addition to breed-specific traits, such as the case of Belgian Malinois, these dogs present very high drives besides being very active when kennelled. A stressful event often initiates clinical signs, a phenomenon often observed in working dogs [2, 6], in contrast to what is observed in sedentary dogs, where exercise can help with chronic diarrhoea [20]. Training and active work are sources of multiple events that can induce stress, thus providing various opportunities to trigger clinical signs. Besides, exercise can also offset a phenomenon similar to runner’s diarrhoea in humans. Decreasing kennel stress and better preparing working animals from a very young age to cope with stress is warranted and may help reduce this problem. Preparing future working dogs for predictable challenges from a very young age has recently been a topic of interest [21, 22]. It may set the stage for study work analysing its effect on this type of diarrhoea.

Adding fiber, and consequently, short-chain fatty acids can help to protect from colitis [6]. It may be an interesting supplementation to regularly maintain working dogs due to their proneness to develop colitis. Still, some difficulties may arise when administering it to dogs. The psyllium presentation used in this study was a powder, and the amount administered to large dogs may decrease appetence and overall palatability. Some cases may also require medication introduction, such as antibiotics (oxytetracycline, metronidazole, or tylosin) [23]. Metronidazole, in particular, is also effective in reducing chronic intestinal inflammation and colitis [24]. No medication was used for any of the dogs enrolled. Probiotics have also been suggested for human athletes, as they can colonize the gastrointestinal tract, adding benefits to health in general, as changes in immune and inflammatory markers in humans [8]. This approach may be of interest to working dogs, so further studies are required to test this possibility.

This study presents some limitations, namely its sample size, the lack of a control group, and the fact that it was not double-blinded. Since the present study showed positive results in managing chronic large-bowel diarrhoea, future studies should enroll a larger number of animals and a control group.

## Conclusions

This study described the effect of psyllium husk in managing chronic large-bowel diarrhoea, with patients showing decreasing defecation frequency, improved stool consistency, and weight gain. Beneficial effects were felt beyond the period of supplementation for most animals. Further studies on alternative approaches to the management of non-responsive cases are required.

## Methods

The study protocol was approved by the ethical review committee of the University of Évora (Órgão Responsável pelo Bem-estar dos Animais da Universidade de Évora). The study took place between January and June 2020. Animals were signaled from the population of police working dogs of the Guarda Nacional Republicana (Portuguese Gendarmerie Canine Unit), based on history, trainer complaints, physical (body weight, body condition score, increased frequency of defecation, soft or liquid stool, excess faecal mucus, slight weight loss, difficulty maintaining an adequate body condition score, continuously or intermittently) and laboratory examination consistent with chronic idiopathic large-bowl diarrhoea. All applicable international, national, and institutional guidelines for the care and use of animals were followed. To be included in the study, animals should be active police working dogs, have a bodyweight ≥15 kg, and age > 2 years. Patients with other illnesses (ruled out through physical examination, complete blood count, and serum biochemistry profile) and on any other treatment protocol were excluded.

Blood samples were collected for the assessment of complete blood count, albumin (ALB), total protein (TP), urea, creatinine (Crea), alkaline phosphatase (ALP), alanine aminotransferase (ALT), bilirubin, cholesterol (Chol), sodium (Na+), potassium (K+), chloride (Cl^−^), calcium (Ca^2+^) and phosphorus (P) (sent to the lab and analysed immediately after collection). Faecal samples were also obtained, and faecal flotations were performed (zinc sulfate faecal flotation). Faecal Clostridium (SBA), clostridial enterotoxin (ELISA), and Salmonella media screening (blood agar, MacConkey’s agar, Hektoen enteric agar, or Campy BAP, then placed in selenite enrichment) were also performed.

All dogs received soluble fiber, psyllium husk, commercially available as a powder, at the dose of 4 tablespoons/day for 1 month [2, 15]. According to the Bristol stool chart, daily registration of faecal characteristics (type, frequency, and color) was maintained during the supplementation month and for an additional month. The same researcher conducted all observations. Animals were weighed weekly. A very good response was considered if faeces were usually normal, with only occasional diarrhea responses, but at a lower frequency than before treatment. The response was deemed good if normal faeces predominated, with common diarrhea episodes, but frequently and less severe. In a poor response, the diarrhoea’s frequency and severity were unaffected by fiber supplementation [2]. The need for any drug to manage diarrhoea was registered.

Each week’s faecal scoring and frequency were compared to the initial evaluation with the Wilcoxon Signed Rank test. The paired-samples t-test was used to compare the body weight of each week with the initial value. A *p* < 0.05 value was set.

## Data Availability

The data that support the findings of this study are available from the Guarda Nacional Republicana, but restrictions apply to the availability of these data, which were used under license for the current study, and so are not publicly available. Data are however available from the authors upon reasonable request and with permission of Guarda Nacional Republicana.

## Abbreviations

ALB: Albumin
ALP: Alkaline phosphatase
ALT: Alanine aminotransferase
Ca^2+^: Calcium
Chol: Cholesterol
Cl^−^: Chloride
Crea: Creatinine
K^+^: Potassium
Na^+^: Sodium
TP: Total protein
P: Phosphorus
